# Anatomical, Molecular–Genetic, and Phytochemical Study of Species from the Genus *Equisetum* in Bulgaria

**DOI:** 10.3390/plants15010016

**Published:** 2025-12-20

**Authors:** Krasimir Todorov, Ginka Antova, Zhana Petkova, Olga Teneva, Maria Angelova-Romova, Rumen Mladenov, Samir Naimov, Elena Apostolova, Donika Gyuzeleva, Tsvetelina Mladenova, Hyulia Panayotova, Plamen Stoyanov

**Affiliations:** 1 Department of Botany and Biological Education, Faculty of Biology, University of Plovdiv “Paisii Hilendarski”, 24 Tzar Assen Str., 4000 Plovdiv, Bulgaria; ktodorov@uni-plovdiv.bg (K.T.); rummlad@uni-plovdiv.bg (R.M.); dgyuzeleva@uni-plovdiv.bg (D.G.); cmladenova@uni-plovdiv.bg (T.M.); stu2505108011@uni-plovdiv.bg (H.P.); pstoyanov@uni-plovdiv.bg (P.S.); 2 Department of Bioorganic Chemistry, Faculty of Pharmacy, Medical University of Plovdiv, Vasil Aprilov Str. 15A, 4002 Plovdiv, Bulgaria; 3 Department of Chemical Technology, Faculty of Chemistry, University of Plovdiv “Paisii Hilendarski”, 24 Tzar Asen Str., 4000 Plovdiv, Bulgaria; zhanapetkova@uni-plovdiv.bg (Z.P.); olga@uni-plovdiv.bg (O.T.); maioan@uni-plovdiv.bg (M.A.-R.); 4 Department of Molecular Biology, Faculty of Biology, University of Plovdiv “Paisii Hilendarski”, 24 Tzar Assen Str., 4000 Plovdiv, Bulgaria; naimov0@uni-plovdiv.bg (S.N.); eapostolova@uni-plovdiv.bg (E.A.)

**Keywords:** *Equisetum*, anatomical features, molecular taxonomic analyses, chemical composition, fatty acids, sterols, phospholipids, tocopherols

## Abstract

Five species of the genus *Equisetum* distributed in Bulgaria were studied: four species from the subgenus *Equisetum* (*Equisetum arvense*, *E. telmateia*, *E. sylvaticum*, and *E. palustre*) and one from the subgenus *Hippochaete* (*E. ramosissimum*). The anatomical, taxonomic, and phylogenetic characteristics of the selected species were established. In species belonging to the subgenus *Equisetum*, the endodermis was arranged in the form of a continuous ring, while in the representatives of the subgenus *Hippochaete*, a two-layered endodermis surrounding each vascular bundle was observed. The results from the DNA barcoding supported the taxonomic treatment of the studied species. The chemical and lipid compositions of the plants were also investigated. The *Equisetum* species had a similar chemical composition and a high content of sterols and phospholipids. In the glyceride oils, palmitic acid predominated, ranging from 69.5% to 78.7%. β-sitosterol was the main component in the sterol fraction, while the tocopherol content was found to be remarkably low in two of the samples (37.6–82.8 mg/kg), with α-tocopherol being predominant. In the phospholipid fraction, the major classes were phosphatidylethanolamine, phosphatidylcholine, phosphatidylinositol, and phosphatidic acids. The chemical composition of the studied species and their high biologically active lipid constituents suggested that they were suitable for application in various directions.

## 1. Introduction

The genus *Equisetum* L. is the only extant genus in the family Equisetaceae, class Equisetopsida. According to Tutin et al. [1], in Flora Europaea, the genus comprises 10 species. These species are distributed in several subgenera, of which *Equisetum* contains eight species and *Hippochaete* eight species [2,3,4,5].

Subgenus *Equisetum* is one of the most known categories of horsetails and includes many representatives such as *E. arvense* L., *E. palustre* L., *E. sylvaticum* L., *E. telmateia*, *E. fluviatile*, and *E. hyemale*, while the subgenus *Hippochaete* is characterized by *E. ramoosissimum* as the main representative.

The habitat of horsetails covers regions from Greenland and Siberia to the temperate zones of tropical America, the Cape of South Africa, as well as South and South-East Asia [6]. Some representatives of horsetails have been found in Bulgaria: *E. hyemale* L., *E. ramosissimum* Desf., *E. fluviatile* L., *E. palustre* L., *E. sylvaticum* L., *E. arvense* L., and *E. telmateia* Ehrh. In addition, data about the following species were reported by Jordanov [7]: *E. sylvaticum* L.; *E. maximum* Lam. (syn. *E. majus*, *E. telmateia*); *E. arvense* L. (syn. *E. arcticum*); *E. palustre* L.; *E. limosum* L. (syn. *E. heleocharis*); *E. ramosissimum* Desf. (syn. *E. elongatum*); and *E. hyemale* L.

The following species have been listed in the *Flora of Bulgaria* [8]: *E. arvense* L., *E. telmateia* Ehrh. (syn. *E. maximum* Lam.), *E. limosum* L. (syn. *E. fluviatile* L., *E. heleocharis* Ehrh.), *E. palustre* L., *E. sylvaticum* L., *E. ramosissimum* Desf., and *E. hyemale* L. In the *Field Guide to the Vascular Plants of Bulgaria* [9], the following species were recorded: *E. sylvaticum* L., *E. arvense* L., *E. telmateia* Ehrh. (syn. *E. maximum*), *E. hyemale* L., *E. ramosissimum* Desf., *E. palustre* L., and *E. fluviatile* L. (syn. *E. limosum*). According to Delipavlov and Cheshmedzhiev [10], the recorded species are *E. telmateia* Ehrh., *E. sylvaticum* L., *E. arvense* L., *E. ramosissimum* Desf., *E. fluviatile* L., *E. palustre* L., and *E. hyemale* L. In the latest edition of the *Determinant of Local and Foreign Higher Plants in Bulgaria* [11], in addition to the previously mentioned species, *E.* × *moorei* Newman is also included.

The plants can reproduce by spores [12]. Subgenus *Equisetum* is marked by species with numerous lateral branches, and in contrast to *Equisetum*, *Hippochaete* plants exhibit no lateral branching [13]. Moreover, compared to other plants, species of the genus *Equisetum* possess appreciated anatomical and morphological features that allow them to sustain mechanical damage when the amount of water in the cells changes during the growing season [14]. There are many studies on the anatomical features of horsetails, showing their value in solving taxonomic problems [15,16,17].

One of the most important features in the anatomical structure of horsetail stems is the mechanical tissue. Some authors classify it as collenchyma [18,19], while others refer to it as sclerenchyma [20,21]. To avoid confusion in the literature, several researchers have proposed neutral terms such as hypodermis [19], hypodermal steroma [22], and reinforcing tissue [23]. The vascular system of horsetails is represented by a siphonostele [16]. The vascular bundles are closed, collateral, positioned opposite the stem ridges, and arranged around a prominent central cavity.

One of the most recent studies on the anatomical characteristics of horsetails is that of Feoktistov and Goreeva [24]. In their publication, the authors present data on the anatomical structures of nine species and five interspecific hybrids, emphasizing key diagnostic features—specifically, the size and position of mechanical tissue within the stem, as well as the location and number of endodermal layers. Investigating the phylogenetic characteristics of horsetails, Christenhusz et al. [25] measured genome sizes across 28 populations representing 18 species. By comparing their findings with previous studies [26,27,28,29], the authors constructed a phylogenetic framework that reflects the evolutionary dynamics of the genome size within the genus *Equisetum*. Based on DNA sequences from two chloroplast markers (rbcL and the trnL-F region), Des Marais et al. [30] re-evaluated the taxonomy and morphological evolution of horsetails (*Equisetum* spp.). Their study provided new insights into the phylogenetic relationships within the genus and contributed to a more refined understanding of species differentiation and evolutionary patterns.

Horsetail species have been integrated into traditional medicine, serving diverse therapeutic purposes [12]. Building upon their widespread distribution and traditional applications, the phytochemical composition of various *Equisetum* species has garnered increasing scientific interest. Data about the chemical composition of *Equisetum* species are limited. Among all types of horsetails, *E. arvense* predominates, due to its medicinal use [31]. Despite the limited data on the overall chemical composition (ash content—22.0% and water content—15.0%) of the species *E. arvense* declared by Sandhu et al. [32] several studies have highlighted the presence of diverse bioactive compounds across different *Equisetum* species. *E. arvense* contains alkaloids, flavonoids, polyphenols, carbohydrates, tannin, proteins and amino acids, phytosterols, saponins, sterols, ascorbic acid, etc. [33]. *E. telmateia* contains kaempferol and quercetin derivatives as well as phenolic acids [34]. Alkaloids, including nicotine and palustrine, have been detected in several species, particularly *E. palustre* [35]. Given the abundance of polyphenols and other bioactive compounds across *Equisetum* species, their physiological relevance has been increasingly explored in cellular and molecular contexts. Experimental evidence indicates that polyphenols may provide indirect protection by activating antioxidant transcription via antioxidant-responsive elements [36]. Furthermore, polyphenols can influence cellular signaling pathways involved in cell proliferation [37] and cell cycle progression [38]. *Equisetum* species also display antioxidant, anti-inflammatory, antiseptic, and anticancer activities [39].

In addition to their antioxidant properties, horsetail species also exhibit a diverse lipid profile. The fatty acid composition of the leaves of four horsetail species was determined by Kokten et al. [40], and the predominant ones identified were linolenic, palmitic, linoleic, hexadecatrienoic, and oleic acids. Minor constituents included palmitoleic, stearic, trienoic, and juniperonic acids, detected at lower concentrations. Other fatty acids—including lauric, myristic, pentadecanoic, hexadecadienoic, margaric, arachidic, gadoleic, and behenic acids—were also detected at concentrations below 1%. Similar trace amounts of these fatty acids have also been reported in other *Equisetum* species by previous studies [41].

Other biologically active substances, such as sterols, were also examined. According to Takatsuto and Abe [42], in the strobilus of *E. arvense*, campesterol (33.8%) and sitosterol (56.5%) were identified as the major ones, while 24-methylenecholesterol (2.0%), 22-dihydrobrassicasterol, and stigmasterol were minor components.

Despite the presence of these previous studies, there has been no extensive research on the anatomical, molecular–genetic, and phytochemical study of species from the genus *Equisetum* from Bulgaria.

For that reason, the aim of this study was to investigate the anatomical, taxonomic, phylogenetic, and phytochemical characteristics of selected *Equisetum* species. Due to the limited data on the chemical and lipid composition of *Equisetum* species in Bulgaria—specifically *E. arvense*, *E. telmateia*, *E. sylvaticum*, *E. palustre*, and *E. ramosissimum*—this research seeks to enhance understanding of the genus *Equisetum*. For the first time, the present study provides a comparative anatomical, molecular–genetic, and phytochemical investigation of *Equisetum* species distributed in Bulgaria. The results obtained may serve as a solid foundation for future research within the genus. This study presents a detailed analysis of the phytochemical constituents and highlights their physiological relevance and potential medicinal applications. Through comparative analysis, the study will identify both shared and unique metabolites among the five species, offering valuable insights into their chemical diversity.

## 2. Results and Discussion

### 2.1. Anatomical Features and Molecular Taxonomic Analyses

The anatomical study of the specified species from the genus *Equisetum* presented in this article is the first of its kind in the country. It provides data that could be used in future taxonomic research within the genus.

Subgenus *Equisetum*

**Figure 1:**
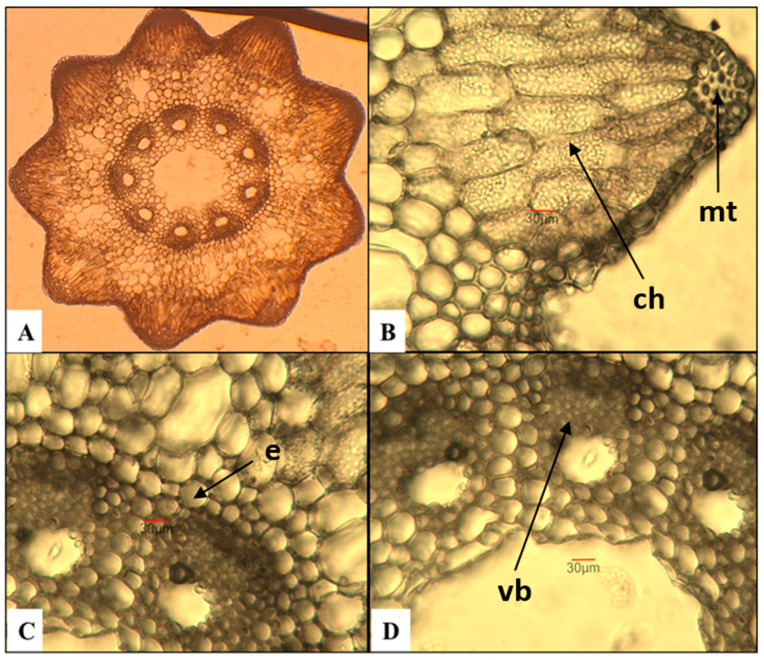
Anatomical features of the stem in *E. arvense*. (**A**)—Cross section of the stem; (**B**)—mechanical tissue (mt) and chlorenchyma (ch); (**C**)—vascular bundles with endodermis (e); (**D**)—vascular bundles (vb) with part of the central cavity of the stem.

*E. arvense*—field horsetail (Figure 1)

The number of ridges on the stem varies between 9 and 10, and they are well-defined. These results contrast with a study by Feoktistov and Gureeva [24], in which the authors report a minimum of 5 ridges, rarely reaching 18. Having analyzed the same species, Pallag et al. [43] established a range of 6 to 18 ridges. The mechanical tissue is well-developed and is located in the region of the ridges beneath the epidermis. The chlorenchyma, composed of three to five layers of cells, is situated beneath it. The endodermis is uniseriate. The number of vascular bundles corresponds to the number of ridges on the stem. The central cavity has an average measurement of approximately 400 µm, which represents about 1/5 of the stem’s diameter. Although it was examined by Feoktistov and Gureeva [24], the authors conclude that it cannot be used as a diagnostic feature for species of the genus *Equisetum*.

**Figure 2:**
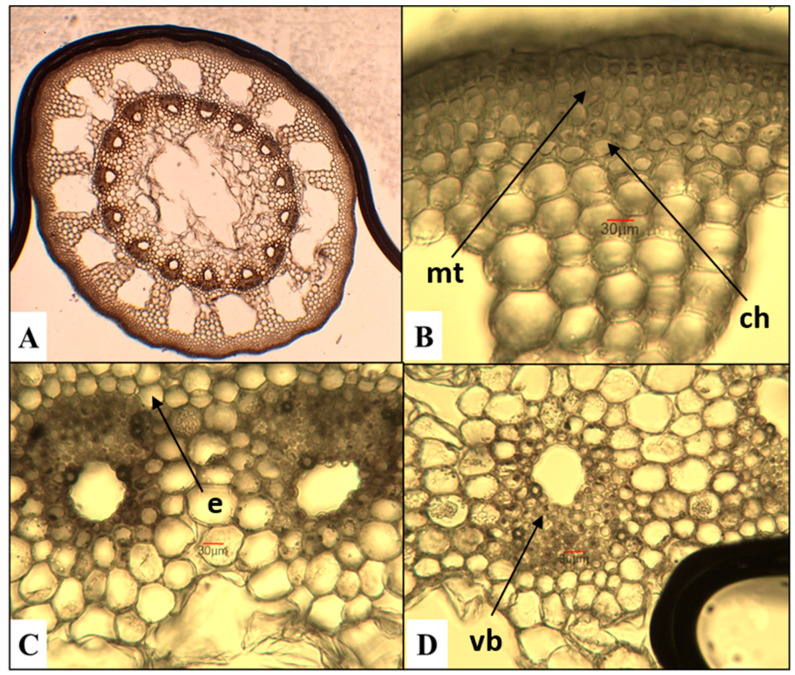
Anatomical features of the stem in *E. telmateia*. (**A**)—Cross section of the stem; (**B**)—mechanical tissue (mt) and chlorenchyma (ch); (**C**,**D**)—vascular bundles (vb) with endodermis (e).

*E. telmateia*—great horsetail (Figure 2)

The cross section of the *E. telmateia* stem reveals a poorly defined multi-ridged structure. The mechanical tissue is poorly developed, consisting of several layers of cells located in the ridged areas. The chlorenchyma tissue is composed of several rows of cells arranged in the form of arc-shaped layers beneath the stem ridges. The endodermis consists of a single layer of cells. The stele comprises vascular bundles surrounded by parenchymatous cells and a central zone characterized by a well-defined cavity.

**Figure 3:**
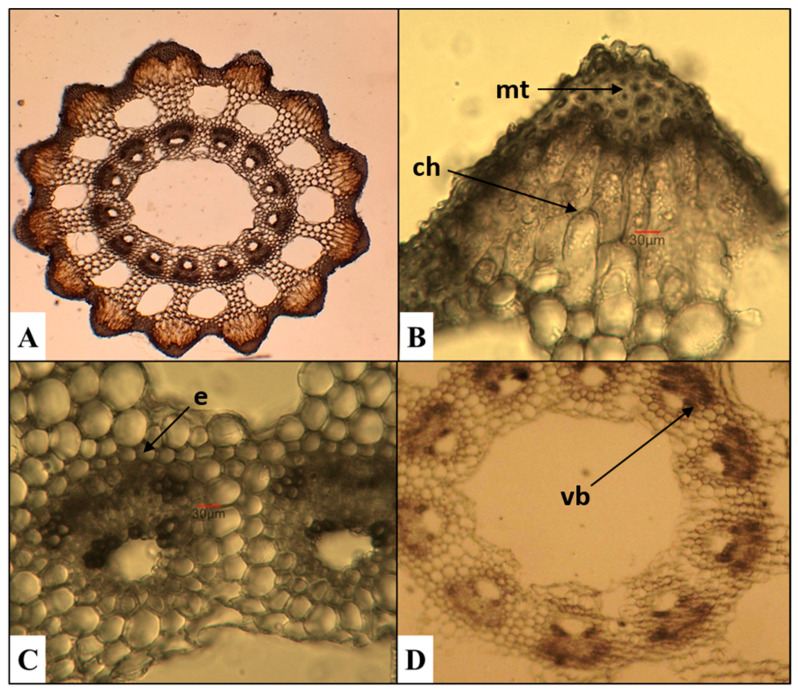
Anatomical features of the stem in *E. sylvaticum*. (**A**)—Cross section of the stem; (**B**)—mechanical tissue (mt) and chlorenchyma (ch); (**C**)—vascular bundles with endodermis (e); (**D**)—vascular bundles (vb) with the central cavity of the stem.

*E. sylvaticum*—wood horsetail (Figure 3)

The ridges on the stem are well-defined, trapezoidal when viewed in a cross section, and 10–14 in number with faintly noticeable spines. The obtained results differ from those of Feoktistov and Gureeva [24], according to whom the number of ridges is 8–16 with clearly visible spines arranged in two rows. The mechanical tissue is multiseriate. Following it, the chlorenchyma can be observed in two–three rows of cells, transitioning into parenchymatous tissue, which encircles cavities equal to the number of ridges. Marin et al. [44] report that the chlorenchyma in *E. sylvaticum* consists of three–four rows of cells in the ridged area, and in one–two layers in the space between them. The endodermis is in the form of a compact ring, surrounding the central part of the stem with the vascular bundles located within it. At the center is the central cavity, which occupies 1/3 of the stem’s diameter.

**Figure 4:**
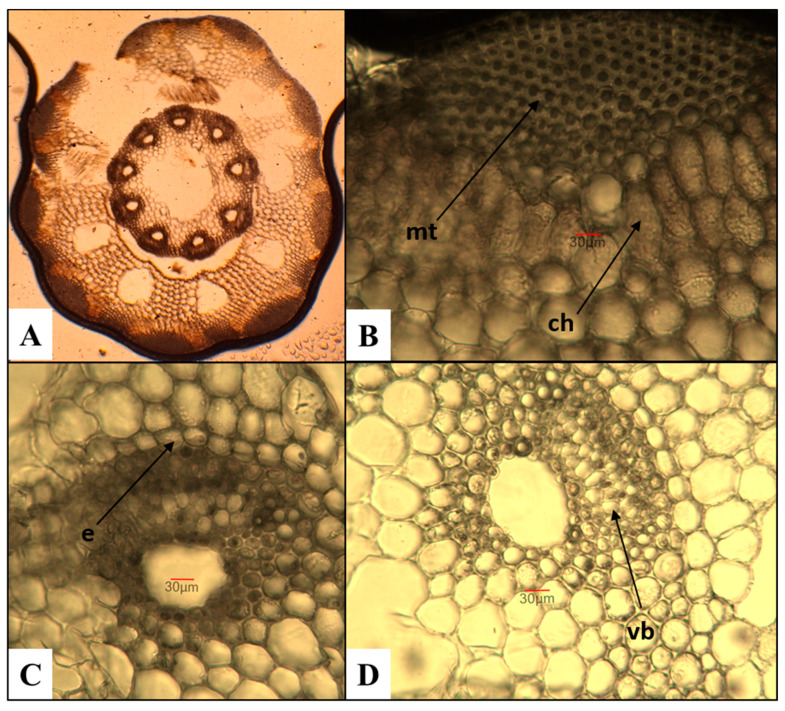
Anatomical features of the stem in *E. palustre*. (**A**)—Cross section of the stem; (**B**)—mechanical tissue (mt) and chlorenchyma (ch); (**C**,**D**)—vascular bundles (vb) with endodermis (e).

*E. palustre*—marsh horsetail (Figure 4)

The stem has well-defined ridges, they are 9–10 in number, triangular when viewed in cross section, and rounded at the apex. The chlorenchyma consists of several rows of cells, located beneath the mechanical tissue in the area of the ridges. The endodermis is arranged as a compact ring around the vascular bundles. Similar to the results published by Feoktistov and Gureeva [24], the central cavity is quite narrow, occupying about 1/4 of the stem’s diameter.

Subgenus *Hippochaete*

**Figure 5:**
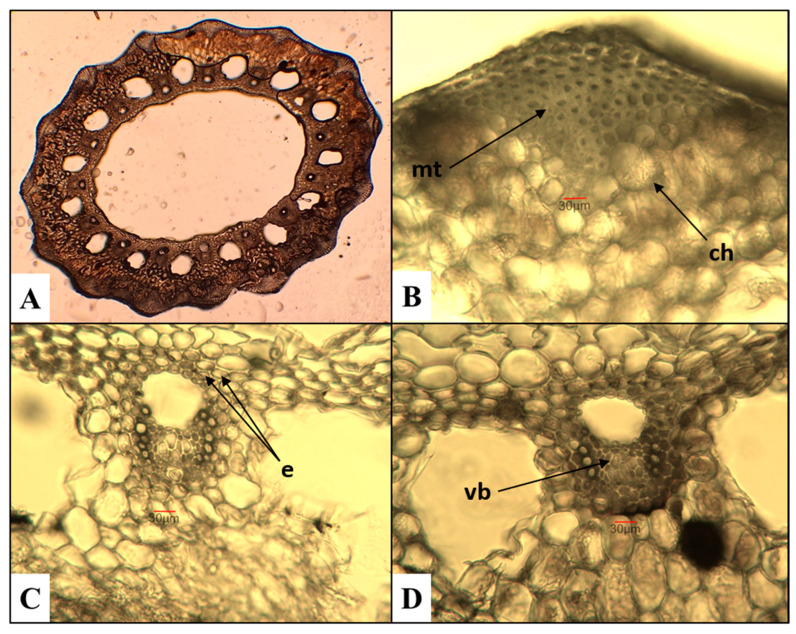
Anatomical features of the stem in *E. ramosissimum*. (**A**)—Cross section of the stem; (**B**)—mechanical tissue (mt) and chlorenchyma (ch); (**C**,**D**)—vascular bundles (vb) with endodermis (e).

*E. ramosissimum*—branched horsetail (Figure 5)

The cross section of the stem shows 15 broadly triangular ridges. In contrast, Feoktistov and Gureeva [24] report that their number varied between 5 and 21, depending on the stem diameter. The arrangement of the mechanical tissue follows the contour of the ridges; it is multiseriate and does not reach the vascular bundles. The cells of the chlorenchyma tissue are arranged in several rows. The endodermis consists of two layers of cells surrounding the vascular bundles. The central cavity occupies 2/3 of the stem’s diameter.

In addition to the endodermis, the anatomical features presented in Table 1 can also be used to distinguish the two subgenera.

For conducting the molecular–taxonomic analyses of the studied species, a single region encoded in the chloroplast genome—tRNA^leu^—was selected. This molecular marker provides a good basis for assessing molecular–taxonomic links and relationships within the genus and species.

**Figure 6:**
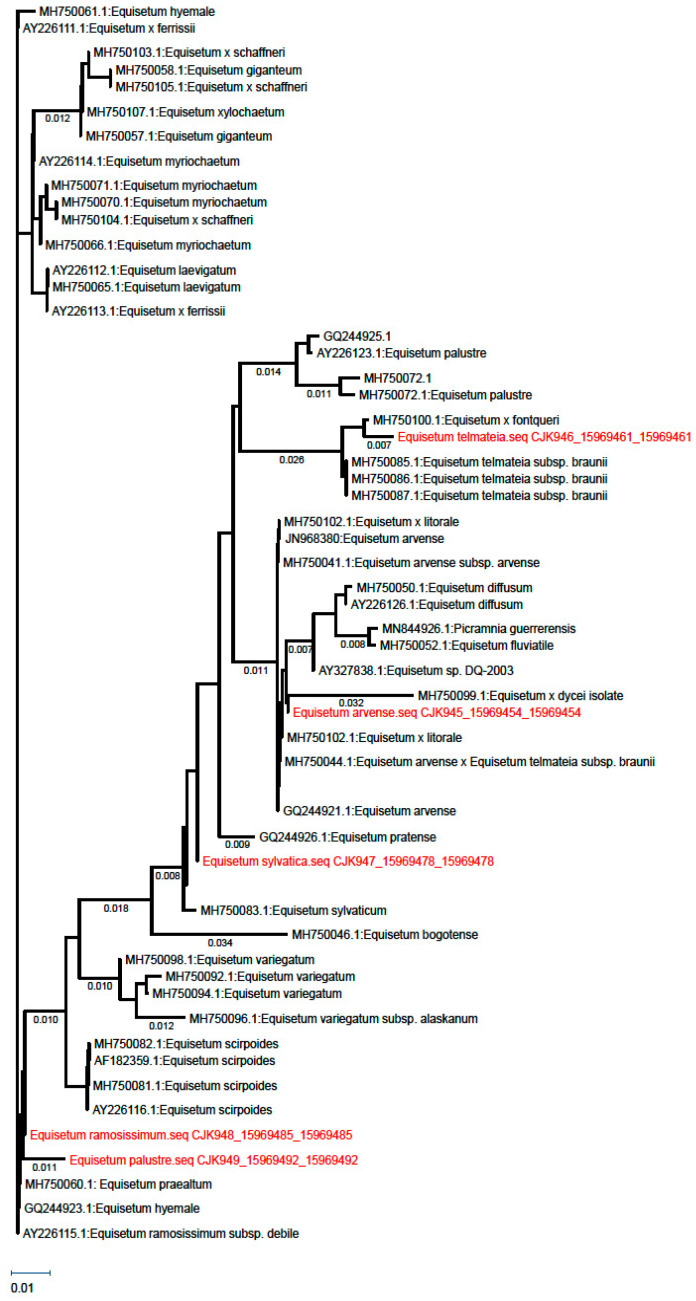
Bayesian phylogenetic tree reconstructed from tRNA^Leu^ sequences.

The Bayesian phylogenetic tree reconstructed from the tRNA^leu^ sequences (Figure 6) reveals two major clades with high posterior probability support (>0.95), corresponding to the subgenera *Equisetum* and *Hippochaete.* Samples of *E. arvense*, *E. telmateia*, *E. sylvaticum*, and *E. palustre* cluster within the *Equisetum* clade alongside conspecific NCBI sequences (e.g., JN968380 *E. arvense*, MH750085 *E. telmateia*), while *E. ramosissimum* groups in the *Hippochaete* clade with *E. hyemale* and related taxa (e.g., MH750061, AY226115). This topology confirms the infrageneric delimitation and placement of Bulgarian populations.

The molecular clustering in Figure 6 aligns with the anatomical distinctions between subgenera. Species in the *Equisetum* clade (*E. arvense*, *E. telmateia*, *E. sylvaticum*, *E. palustre*) share a uniseriate endodermis forming a continuous ring around vascular bundles, well-defined ridges (9–14), and multiseriate mechanical tissue beneath the epidermis. In contrast, *E. ramosissimum* in the *Hippochaete* clade exhibits a two-layered endodermis per vascular bundle, broader triangular ridges (15), and mechanical tissue not reaching bundles—features consistent across both datasets and supporting subgeneric separation without notable homoplasy. This congruence validates an integrative taxonomy for *Equisetum* in Bulgaria.

### 2.2. Chemical Composition of Different Species of Equisetum

The main compounds of the chemical composition of the studied species of *Equisetum* (protein, carbohydrates, glyceride oil, insoluble fibers, mineral content and moisture) are presented in Table 2.

The protein content in the examined samples ranged from 9.3% (*E. telmateia*) to 15.9% (*E. sylvaticum*). The studied species had a high carbohydrate content, ranging from 49.8% (*E. ramosissimum*) to 59.6% (*E. sylvaticum*). The fiber content in the samples ranged from 20.1% (*E. telmateia*) to 31.4% (*E. arvense*). The analyzed aerial stems of the species exhibited low lipid content: 1.9% (*E. sylvaticum*), 2.7% (*E. telmateia* and *E. ramosissimum*), 3.5% *(E. palustre*), and 3.7% (*E. arvense*). The studied *Equisetum* species had a high mineral content—from 12.3% (*E. sylvaticum*) to 24.0% *(E. ramosissimum*). The moisture content ranged from 10.3% (*E. sylvaticum*) to 15.0% (*E. arvense*), and these values were close to those determined by Sandhu et al. at 15% [32] and Gua et al. at 8.85% [45] for *E. arvense*.

Similar results for the crude protein content (15.32%) were reported in studies on the chemical composition of *E. arvense* [45]. The content of carbohydrates in *E. arvense* was within the content reported by Gua et al. (53.2%) [45]. The glyceride oil content obtained for *E. arvense* was higher than the 2.21% reported by Goa et al. for the same species prevalent in Korea [45]. However, there were also reports of significantly higher fat content (5.4%, 4.1%, and 4.6%) in the aerial parts of *E. fluviatile* grown in James Bay Lowland, Canada, during early spring, summer, and autumn [46]. Paine et al. [47] studied Himalayan horsetails species *E. diffusum*, grown at different altitudes in the Eastern Himalayas, and found lipid contents ranging from 44.58 mg/g dry weight (dw) (4.46%) to 120.83 mg/g dw (12.08%); these values were also higher than the present data. The values for the ash content corresponded with the data on the total ash content of *E. arvense* (15.3%, 16.8%, 17.2%, 20.42%, and 22.0%) [32,45,46]. Thomas and Prevett [46] studied the chemical composition of the aerial parts of *E. fluviatile* during early spring, summer, and fall. During the study period, the protein content decreased from 14.0% to 7.3%, and a reduction in the water content from 87.6% to 84.9% was observed. While the lipid content decreased from 5.4% to 4.1% in the first period, it later increased to 4.6%. A similar trend was noted in the ash content: 15.3% (spring), 17.2% (summer), and 16.8% (autumn).

The caloric value of the examined samples ranged from 273.9 kcal/100 g (*E. ramosissimum*) to 319.1 kcal/100 g (*E. sylvaticum*). Compared to wheat (334 kcal/100 g), sorghum (343 kcal/100 g), dry beans (341 kcal/100 g), soyabeans (335 kcal/100 g), and quinoa (342 kcal/100 g), the studied species had a lower energy value, though it was similar to that of dried mushrooms (296 kcal/100 g), walnuts (289 kcal/100 g), and hazelnuts (291 kcal/100 g) [48].

### 2.3. Free Sugar Composition

Plants contain different types of sugars, which are a source of energy and play a role in growth and defense. Significant variations have been observed in the quantity of these components in different organs as well as in different species. The specific types and amounts of sugar vary depending on the plant species, and environmental factors, such as drought or salinity can also affect the sugar content, as plants use sugars to cope with stress [49,50].

The results for the free sugar composition of the studied species are shown in Table 3.

The total free sugar content in the studied species was relatively high (4.32–17.09 g/100 g dw). In general, the maximum content of total sugars was observed in *E. palustre* (17.09 g/100 g dw), followed by *E. ramosissimum* (14.36 g/100 g dw), and minimum sugars were recorded in *E. sylvaticum* (4.32 g/100 g dw), followed by *E. telmateia* (6.10 g/100 g dw) and *E. arvense* (6.14 g/100 g dw). The results indicated the presence of mannose and fructose in all *Equisetum* species studied. The amount of mannose (2.52–8.20 g/100 g dw) was higher than that of fructose (0.85–3.23 g/100 g dw). The species *E. ramosissimum* had the highest content of these sugars, respectively, 8.20 g/100 g mannose and 3.23 g/100 g fructose. Rhamnose content was found in the sugars of the species *E. sylvaticum* (0.95 g/100 g dw) and *E. palustre* (3.28 g/100 g dw), while galactose was found in the species *E. palustre* (3.78 g/100 g dw) and *E. ramosissimum* (2.93 g/100 g dw). Sucrose content was found only in the composition of the sugars of *E. telmateia* (1.04 g/100 g dw). Studies by Paine et al. [47] on Himalayan horsetails show a soluble sugar content of 145 to 175 mg/g (14.5–17.5%), values close to those obtained in the present study for the species *E. palustre* and *E. ramosissimum*. Results that differ from ours were obtained by Gua et al. [45], who found mannose and glucose in the monosaccharide composition of *E. arvanse*, in amounts of 1616.31 mg/100 g (1.62 g/100 g) and 13,351.88 mg/100 g (13.35 g/100 g), respectively.

### 2.4. Lipid Composition

Lipids are both products and functional components of living cells. They are primarily composed of triacylglycerols, accompanied by substances such as sterols, tocopherols, and phospholipids. The latter are bioactive compounds that can exert pharmacological effects on humans and animals. Data on the content of biologically active substances in glyceride oils and in the five species of *Equisetum* are provided in Table 4.

The amount of unsaponifiable matter in the studied *Equisetum* species oils ranged from 16.1% (*E. ramosissimum*) to 31.1% (*E. sylvaticum*). The results were in good agreement with the amount generally reported in crude vegetable oils, according to the *Codex Alimentarius* (≤9.0–28.0%) [51]. The sterol content in the oils varied between 1.3% (*E. telmateia*) and 3.0% (*E. arvense*), while quantities of sterols in the plant were significantly lower—between 0.04 and 0.11%. Tocopherols (vitamin E) were found only in oils isolated from two species of horsetail *E. palustre* and *E. ramosissimum*, with the tocopherol content found to be remarkably low, ranging from 37.6 to 82.8 mg/kg (in the plants—2.90 and 1.02 mg/kg, respectively). These values were comparable to those reported for coconut oil (0–50 mg/kg) and palm kernel olein and stearin (0–90 mg/kg and 0–89 mg/kg, respectively) [51]. The dominant tocopherol isomer in the studied oils from two species (*E. palustre* and *E. ramosissimum*) was α-tocopherol (100%). The individual tocopherol composition of these oils was similar to the tocopherol profile of the most common vegetable oils—such as sunflower and safflower, where α-tocopherol predominated (89–2468 mg/kg) [51].

The highest phospholipid content was observed in the oils of species *E. sylvaticum* (11.1%) and *E. palustre* (15.3%), and 0.21% and 0.54% in the plants, respectively. In comparison, the phospholipid content in the oils of species *E. telmateia* and *E. ramosissimum* was 6.3% and 6.7%, and in the plants—0.17% and 0.18%, respectively. The minimum phospholipid content was noted in *E. arvense* (1.6% in the oil and 0.06% in the plants). The total phospholipid content was higher than that found in vegetable oils, where it typically ranged between 1.0% and 1.5% [51,52]. The studied species had low oil content (1.9–3.7%) but were rich in biologically active compounds as well as sterols and phospholipids. This sparked scientific interest in exploring the fatty acid, sterol, and phospholipid composition of aerial parts of these five *Equisetum* species.

#### 2.4.1. Fatty Acid Composition

Table 5 shows the fatty acid composition of the glyceride oils from the studied *Equisetum* species.

In the oils of the four species (*E. arvense*, *E. telmateia*, *E. sylvaticum*, and *E. palustre*) palmitic acid predominated, ranging from 69.5% (*E. sylvaticum*) to 78.7% (*E. arvense*), followed by myristic acid, which varied from 5.2% (*E. telmateia*) to 11.5% (*E. palustre*). These were followed by oleic acid (1.4% in *E. arvense* to 7.2% in *E. sylvaticum*), gadoleic acid (2.1% in *E. sylvaticum* to 3.2% in *E. palustre*), and lignoceric acid (1.5% in *E. arvense* to 5.1% in *E. sylvaticum*). Linoleic acid was present in amounts from 0.8% (*E. telmateia*) to 4.5% (*E. palustre*), and erucic acid was from 1.0% (*E. arvense* and *E. palustre*) to 3.3% (*E. sylvaticum*). In the fatty acid profile of species *E. ramosissimum*, palmitic acid was dominant (70.0%), followed by linolenic (10.6%), gadoleic (9.0%), and oleic acid (4.5%). The oils of the studied *Equisetum* species also contained a trans isomer of oleic acid—elaidic acid, which was present in amounts of 0.9–1.5% in the oils of three *Equisetum* species (*E. arvense*, *E. telmateia*, *E. sylvaticum*), while in the oils from the other two types (*E. palustre* and *E. ramosissimum*), the trans acid content was very low—0.1%. The lipids of the studied *Equisetum* species were not rich in essential n-6 and n-3 fatty acids.

The highest content of essential linoleic acid (n-6) was found in the species *E. ramosissimum* (10.6%), followed by the species *E. palustre* (4.5%). In the remaining three species, its content varied from 0.8 to 1.6%. Linolenic acid (n-3) was present in minimal amounts (0.1%) in the oils of only two of the studied *Equisetum* species (*E. palustre* and *E. ramosissimum*).

Similar results to ours were obtained by Paine et al. [47] in their study of the fatty acid composition of neutral lipids isolated from Himalayan horsetail species *E. diffusum*, grown at different altitudes. In neutral lipids, the total saturated fatty acid content was noticeably high (74.36–95.65%), which was primarily due to the predominance of palmitic acid (C_16:0_)—65.78–84.57%. The palmitic acid content was found to be the highest in the sample from altitude 1980 m (84.57%), which was significantly higher than the samples of higher altitudes (2510 m)—65.78%, while for a sample from the lowest altitude (1390 m), it is 68.07%. In contrast, the linolenic acid (C_18:3_ n-3) content was much lower—1.39% (1980 m), 13.47% (1390 m), and 18.58% (2510 m).

The data obtained in the current study differed from those of other authors who had studied the fatty acid composition of glyceride oils from different *Equisetum* species. Authors from Turkey studied the fatty acid composition of various types of horsetails (*E. arvense*, *E. palustre*, *E. ramosissimum*, and *E. telmateia*) and found that the predominant acid was linolenic acid (41.57–42.57%), followed by palmitic acid (25.93–26.22%) and linoleic acid (8.35–8.92%) [40]. Nokhsorov and Protopopov [53] also found that the main components of total lipids from *E. arvense* growing in the Yakutia are omega-3 (n-3) and omega-6 (n-6) polyunsaturated fatty acids (PUFA), such as linoleic (C_18:2_(n-6)—6.64%) and α-linolenic (C_18:3_(n-3)—44.53%), the contents of which reached 51.17% of the total sum of FA, and saturated fatty acids (SFA) were mainly represented by palmitic acid (C_16:1_–29.21%). Dudareva et al. [41], when studying the fatty acid composition of oils from aerial parts of three horsetail species growing in Central and Northern Yakutia (*E. arvense*, *E. variegatum* and *E. scirpoides*), also found that linolenic (25.2–42.3%), linoleic (8.2–23.4%), and palmitic (22.1–26.8%) acids predominated in their composition. Gua et al. [45] studied the fatty acid composition of *E. arvense* oil and found that α-linolenic (42.83%), linoleic (23.60%), and oleic (7.60%) acids predominated among the unsaturated fatty acids (UFA), and palmitic (19.0%) and stearic (2.47%) acids predominated among the saturated fatty acids. Nokhsorov et al. [54] studied the seasonal changes in the lipid composition of two species of horsetail, *E. variegatum* and *E. scirpoide*, growing in the permafrost zone (Northeastern Yakutia, the Pole of Cold of the Northern Hemisphere), with average daily air temperatures in summer of +17.8 °C, in autumn of +0.6 °C, and in winter of −46.7 °C. It was found that the content of linolenic acid in the autumn period decreased compared to the summer season from 39.5% to 25.7%, and in the winter, it increased to 31.5%, respectively, while the amount of saturated palmitic acid increased from 24.7% (summer period) to 30.6% (winter period). The differences in the fatty acid composition of the glyceride oil from different *Equisetum* species are probably due to several factors: their origin, species, biological and ecological conditions, and the altitude of growing, which may affect the synthesis and accumulation of fatty acids in the plants. According to Kokten et al. [40], the predominance of certain fatty acids is not constant even for the same species, because it is influenced by the specific differences in their varieties, as well as the particular climate and especially the temperature of the habitat.

**Figure 7:**
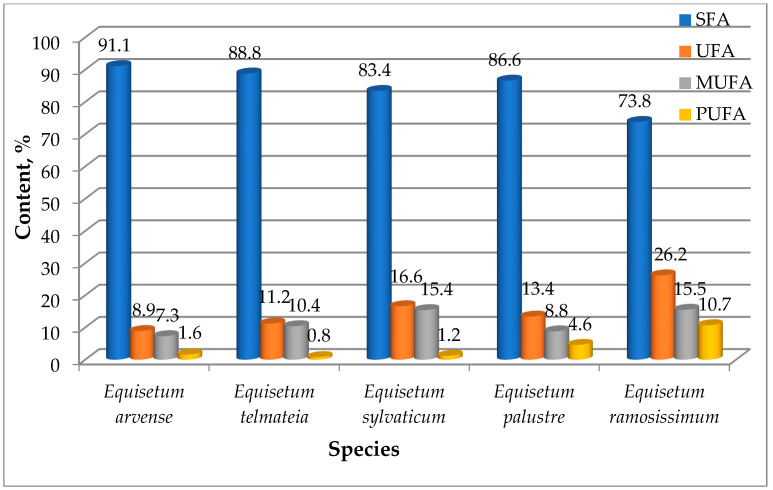
Saturated (SFA), unsaturated (UFA), monounsaturated (MUFA), and polyunsaturated (PUFA) fatty acids in oils from the studies *Equisetum* species.

Figure 7 shows that the amount of saturated fatty acids exceeds 73.0%. The lowest amount of saturated fatty acids was in the oil from the species *E. ramosissimum* (73.8%), and the highest was in oil isolated from the species *E. arvense* (91.1%). The oils from the species *E. telmateia*, *E. sylvaticum* and *E. palustre* contained saturated fatty acids in the following amounts: 88.8%, 83.4%, and 86.6% respectively.

The content of monounsaturated fatty acids (7.3–15.5%) was higher than that of polyunsaturated ones (0.8–10.7%). The amount of mono- and polyunsaturated fatty acids was the highest in the oil from the *E. ramosissimum* species (15.5 and 10.7%) and the lowest in the oil isolated from the *E. arvense* species (7.3 and 1.6%). In the glyceride oils of the *E. sylvaticum* and *E. telmateia* species, the amount of monounsaturated fatty acids (15.4% and 10.4%) was significantly higher than that of polyunsaturated fatty acids (1.2% and 0.8%), while in the *E. palustre* species, the amounts of monounsaturated ones (8.8%) were about twice as high as the polyunsaturated (4.6%). Our results were consistent with the literature data on the fatty acid composition of the neutral lipids of the horsetails samples collected from three altitude zones of Eastern Himalayan region, where the quantity of saturated fatty acids ranged from 74.36% to 95.65%. The primary saturated fatty acid was palmitic acid (C_16:0_)—65.78–84.57%. The content of monounsaturated fatty acids was relatively low—between 1.78% and 4.49%. The authors found that the content of polyunsaturated fatty acids (PUFA) was the highest in the sample from an altitude of 1390 m (21.52%), which was significantly higher than that from an average altitude of 1980 m (2.57%), while for the sample from the higher altitude (2510 m), it was 21.16%. They demonstrated that variation in the MUFA content based on altitude was not visible, whereas variation in the SFA and PUFA content was quite apparent [47].

#### 2.4.2. Sterol Composition

The main components of sterols were β-sitosterol (from 51.1% in *E. arvense* to 69.0% in *E. sylvaticum*) and campesterol (from 24.1% in *E. sylvaticum* to 37.8% in *E. ramosissimum* species) (Table 6).

The lipids of four of the studied species did not contain cholesterol, except for those of the species *E. arvense*, which contained a minimal amount—0.2%. According to the literature data, the cholesterol content in the sterol fraction of vegetable oils varied from trace amounts up to 6.7% [51]. The lipids of *E. arvense* contained a high amount of Δ^7^-stigmasterol—10.4% compared to lipids from other studied species (from 0.7 to 2.2%). In the studied samples, the stigmasterol content was from 0.3 to 3.8%, which was close to its content in sterols of almond (0.4–4.0%) and hazelnut (0–2%) oil. The Δ^5^-avenasterol content (0.2–1.8%) in the studied *Equisetum* species was comparable to its content in sterols of high oleic palm oil (0–1.9%) [51]. The remaining types of sterols ranged from 0.1% to 1.5%. The sterol composition of the *Equisetum* species oils analyzed in this study was consistent with the data reported by Takatsuto and Abe [42] on the sterol profile of lipids isolated from strobilus of *E. arvense*, where β-sitosterol was the predominant component (56.5%), followed by campesterol (33.8%).

#### 2.4.3. Phospholipid Composition of Lipids

The individual composition of the phospholipids in the studied *Equisetum* species was also investigated. In the phospholipid fraction of oils from different samples, major classes of phospholipids were identified (Table 7), particularly phosphatidylethanolamine, phosphatidylcholine, phosphatidylinositol, and phosphatidic acids.

The highest content of phosphatidylethanolamine was found in the lipids of the species *E. arvense* (35.9%) and *E. palustre* (24.3%), and a lower content was observed in the lipids from *E. telmateia* and *E. sylvaticum* (11.7 and 11.6%). The lipids of the species *E. telmateia*, *E. sylvaticum*, *E. palustre*, and *E. ramosissimum* showed a higher content of phosphatidic acids (26.6, 46.0, 42.4, and 30.7%, respectively), while in species *E. arvense* it was 13.2%. Phosphatidylcholine was present in higher amounts in the lipids of *E. sylvaticum* (21.8%) and of *E. palustre* (19.6%), and in other species its amount varied from 12.4% (*E. arvense*) to 15.8% (*E. ramosissimum*). A higher content of phosphatidylinositol was also noted in lipids from *E. telmateia* (23.1%), and a lower content was observed in the lipids from *E. palustre* (5.3%). Monophosphatidylglycerol in the lipids of all the studied samples ranged between 2.6 and 9.4%. The highest levels of lysophosphatidylethanolamine were observed in the lipids from *E. arvense* (17.2%), *E. telmateia* (15.3%), and *E. ramosissimum* (12.3%), while in species *E. sylvaticum* and *E. palustre*, it was 5.5% and 3.6%, respectively. The data indicated that the phospholipids in the different species had a similar qualitative but different quantitative composition. Similar to our results were those of Nokhsorov et al. [54], who studied the seasonal variability of phospholipid composition in the aerial parts of two species of horsetail *E. variegatum* and *E. scirpoides*, growing in the Northeastern Yakutia. They found that phosphatidic acids (PA) predominated in the phospholipid fraction isolated from *E. variegatum* during the summer, autumn, and winter periods. In the phospholipids of *E. scirpoides* during the summer and autumn periods, phosphatidylcholine predominated, while during the winter period, phosphatidic acids were highest. Both plants accumulated PA in response to the decrease in temperature and light in the autumn–winter period. Other phospholipid classes demonstrated different seasonal trends in the studied samples. Phosphatidylcholine was accumulated in *E. variegatum* in the winter period, whereas, in *E. scirpoides*, the content of this phospholipid increased in autumn but sharply decreased in the winter. The content of phosphatidylethanolamine decreased in both plants in winter as compared to the autumn values, but the summer values of this phospholipid were different for two species—in *E. variegatum*, very low values, while in *E. scirpoides*, relatively high values. The differences in phospholipid composition between *Equisetum* species most likely can be explained by their different habitats, different soil and climatic conditions, genetic variability, physiological needs, etc. [54].

## 3. Materials and Methods

### 3.1. Materials

**Figure 8:**
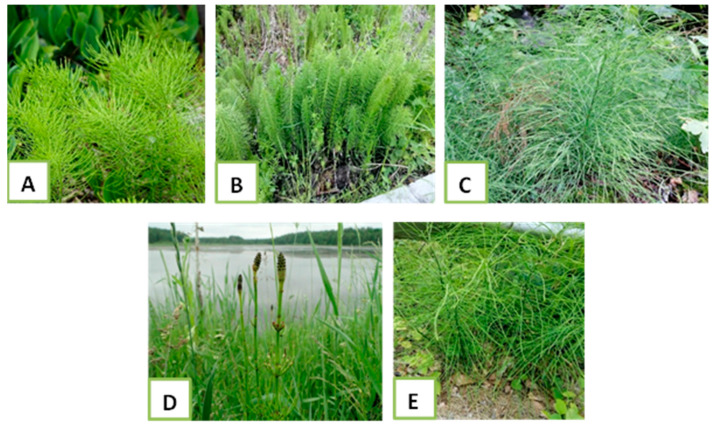
Authors’ photo examples of the examined species. (**A**)—*E. arvense*, (**B**)—*E. telmateia*, (**C**)—*E. sylvaticum*, (**D**)—*E. palustre*, (**E**)—*E. ramosissimum*.

For the present study, use was made of plant material of five *Equisetum* species distributed in Bulgaria (Table 8, Figure 8). The materials were collected and processed during the growing seasons of 2023 from the Rhodope Mountains (Western) region.

### 3.2. Anatomical and Molecular Taxonomic Analyses

For the anatomical analysis of the stems of the studied *Equisetum* species, the classical method was applied [55]. The anatomical analyses were conducted on cross sections from the middle part of the stem in the region of the internodes. For the anatomical studies, the plant parts were preliminarily fixed in 70% ethanol. The following anatomical characteristics were examined, some of which, according to Minarchenko et al. [56], can be used as diagnostic features for distinguishing between species: number of stem ridges; location of the mechanical tissue; location and number of chlorenchyma layers; number of endodermal layers; and dimensions of the central cavity.

The molecular–genetic analysis was performed on fresh plant material. For the molecular analyses, total plant DNA extraction was performed using a kit DNeasy Plant Mini Kit (QIAGEN, Hamburg, Germany), following the procedure specified by the manufacturer. The concentration and quality of the isolated DNAs were determined fluorometrically (Qubit 4 Fluorometer, Thermo Fisher, Waltham, MA, USA) and by 1% agarose gel electrophoresis. To perform the phylogenetic analysis, the target DNA sequence was amplified using PCR. The tRNA^leu^ amplification was performed using forward primer trnL-c (5′-CGAAATCGGTAGACGCTACG-3′; Code B49317) and reverse primer trnL-f (5′-ATTTGAACTGGTGACACGAG-3′; Code A50272). Conventional PCR was performed in 20 µL reactions containing 1 µL template DNA, 0.2 µM of each primer, 1 U Taq DNA polymerase, 0.2 mM of each dNTP, and standard buffer (10 mM Tris-HCl, pH 9.0, 50 mM KCl, 1.5 mM MgCl_2_) using a Biosystems 2720 Thermal Cycler. The cycling conditions were 94 °C for 4 min; 30 cycles of 94 °C for 30 s, 56 °C for 30 s, and 72 °C for 40 s; and a final extension at 72 °C for 7 min. The resulting products were separated on a 1% agarose gel solution. Staining of the gels was carried out using ethidium bromide. All gels were visualized and recorded using a BIO-VISION+3026.WL gel documentation system. The size of the amplified fragments was determined relative to a standard DNA marker —Fermentas GeneRuler SM#0331. For the extraction of PCR products from the gel, a kit from Qiagen, Germany (QIAquick PCR & Gel Cleanup) was used, following the protocol recommended by the manufacturer. After purification, the products were subjected to Sanger sequencing in both directions to ensure the accuracy of the obtained sequences. The quality of the DNA sequences was verified using the Chromas v.2.6.6 software (Technelysium Pty Ltd., Brisbane, Australia). In parallel, questionable nucleotides were reviewed and corrected based on the available chromatograms. In the next stage of the analysis, the resulting consensus sequences were subjected to a BLAST analysis (Basic Local Alignment Search Tool, version 2.15.0), and the sequences most closely related to them were organized into a local database of tRNA^leu^ sequences. Subsequently, the respective sets of sequences underwent multiple comparative analysis using the Megalign package from the Lasergene 10 suite. The Tamura–Nei algorithm, integrated into the program, was used for statistical processing. The absence of nucleotides in certain sequences was integrated into the analysis using the global gap removal model. The results obtained were visualized in the form of a cladogram.

Light microscope images of the examined anatomical features of the stems were taken with a Magnum T microscope(Medline Scientific c/o Calibre Scientific, Rotherham, UK), equipped with a Si5000 photo documentation system, at magnifications of ×100 to ×400, at the Department of Botany and Biological Education, Faculty of Biology, University of Plovdiv “Paisii Hilendarski”. The cross sections were prepared using razor blades.

### 3.3. Chemical Composition

The aerial parts of the plants were used for the phytochemical investigations. All of the analyses were carried out with air-dried material.

The lipids were isolated from 50 g of samples with n-hexane in a Soxhlet extractor [57]. The Kjeldahl method was used for the determination of the total protein content in accordance with the AOAC [58]. The total carbohydrate content was calculated: 100 − (weight in grams [protein + lipids + moisture + ash] in 100 g of dry seeds) [59]. The content of insoluble fibers, ash, and moisture were determined according to the AOAC [58]. The energy value (EV, kcal/100 g) was calculated using the following formula [59]:

EV, kcal/100 g (kJ/100 g) = % proteins × 4.0 (17) + % carbohydrates × 4.0 (17) + % lipids × 9.0 (38).

### 3.4. Soluble Sugars

The composition of the water-soluble carbohydrates was determined through high performance liquid chromatography (HPLC). The sample preparation was described by Georgiev et al. [60]. In brief, 5 g of the ground material was extracted with 100 mL of 60 % ethanol in a shaking water bath at 83 °C for 20 min. The solution was cooled and filtered through Whatman N4 paper, and additional filtration was carried out through a Sep-Pak C18 cartridge (Waters, Milford, MA, USA) and a 0.22 μm membrane filter (Sterivex™-GS Millipore^®^). The determination of the individual sugars was performed on an Agilent^®^ LC 1220 instrument (USA) with a Zorbax Carbohydrate column (150 mm × 4.6 mm, pore size: 70 Å, particle size: 5.0 µm, Agilent, Santa Clara, CA, USA), a Zorbax Reliance Cartridge guard-column (Agilent), and a refractive index detector (RID 1260). The mobile phase (flow rate of 1.0 mL/min) was acetonitrile/water (AcN/H_2_O) (80/20). The temperature of the column and detector was 30 °C. All sugars were identified by comparing the retention times of the analytes with analytical grade standards (glucose, fructose, galactose, rhamnose, xylose, arabinose, sucrose, and maltose (Sigma-Aldrich (Steinheim, Germany)).

### 3.5. Lipid Composition

#### 3.5.1. Fatty Acids

The composition of the fatty acids was determined by gas chromatography (GC) [61]. Fatty acid methyl esters (FAMEs) were obtained by trans-esterification of the glyceride oil with methanol in the presence of sulfuric acid [62]. The determination was carried out on an HP 5890 gas chromatograph (Hewlett–Packard GesmbH, Vienna, Austria) equipped with a capillary column Supelco (Supelco, Bellefonte, PA, USA) of 30 m × 0.25 mm × 0.2 µm (film thickness) and a flame ionization detector (FID). The conditions were as follows: the starting column temperature was 70 °C (for 1 min), increasing at 6 °C/min to 190 °C (3 min), followed by an increase at 10 °C/min to 240 °C. The temperature of the injector and detector was 250 °C. Identification was performed by comparison of the retention times of the unknown peaks with the retention time of a standard mixture of FAMEs (Supelco, 37 comp. FAME mix., Bellefonte, PA, USA). The software used was Data Apex Clarity TM 2.4.1.93/2005 (DataApex, Prague, Czech Republic).

#### 3.5.2. Sterols

The isolated glyceride oils (3–5 g) from the samples were subjected to saponification with 2 N KOH, and the unsaponifiable matter was extracted with hexane [63]. The total sterols were isolated from the unsaponifiable matter through thin-layer chromatography, and their content was determined spectrophotometrically at 597 nm [64]. The sterol composition was determined on an HP 5890 gas chromatograph (Hewlett–Packard GesmbH, Vienna, Austria) with a capillary column DB 5 (25 m × 0.25 mm × 0.25 µm (film thickness), Agilent Technologies, J&W Scientific products Proudly, Santa Clara, CA, USA) and FID. The operating conditions were as follows: temperature starting from 90 °C (3 min) up to 290 °C at a rate of 15 °C/min, and then, the temperature increased up to 310 °C at a rate of 4 °C/min (hold for 10 min). The detector temperature was 320 °C, and the injector temperature was set at 300 °C. The carrier gas was hydrogen. The individual sterol composition was determined by a comparison of the retention times with a standard mixture of sterols [65]. The software used was Data Apex Clarity TM 2.4.1.93/2005 (DataApex, Prague, Czech Republic).

#### 3.5.3. Tocopherols

High-performance liquid chromatography (HPLC) was used for the determination of the tocopherol composition. The equipment was a Merck–Hitachi (Burladingen, Germany) with a florescence detector F-1050 (Merck-Hitachi, Burladingen, Germany) and a D-2500 Merck–Hitachi chromato-integrator (Merck, Darmstadt, Germany). The experimental conditions were fluorescent detection at 295 nm excitement and 330 nm emission, and the column used was Nucleosil Si 50-5 column (250 mm × 4 mm, Merck, Darmstadt, Germany). The mobile phase was hexane: dioxane, 96:4 (*v*/*v*), at a flow rate of 1 mL/min [66]. The individual tocopherols were identified by comparing the retention times with those of the standard reference materials.

#### 3.5.4. Phospholipids

The phospholipid classes were isolated by two-dimensional thin-layer chromatography [67]. The spots of the individual phospholipids were scraped and mineralized with perchloric and sulfuric acid (1:1, *v*/*v*), and their content was determined spectrophotometrically at 700 nm [68].

### 3.6. Statistical Analysis

The results are expressed as the mean values of three parallel determinations ± standard deviations (*n* = 3). Data were analyzed using one-way ANOVA followed by Duncan’s test for multiple comparisons (*p* < 0.05).

## 4. Conclusions

The anatomical analysis of stems identified the endodermis as the most important characteristic distinguishing the two subgenera. In the species belonging to the subgenus *Equisetum* (*E. arvense*, *E. palustre*, *E. sylvaticum*, and *E. telmateia*), the endodermis is arranged in the form of a continuous ring. In representatives of the subgenus *Hippochaete* (*E. ramosissimum*), one can observe a two-layered endodermis surrounding each vascular bundle. Other important anatomical features that can be differentiated between the two subgenera include the number and shape of the stem ridges in cross section and the location of the mechanical tissue. When comparing the obtained results with those of major previous studies by Pallag et al. [42] and Feoktistov and Gureeva [24], differences were observed regarding the number of stem ridges. As for the diameter of the central cavity, our findings support the conclusion of Feoktistov and Gureeva [24] that this feature cannot be used as a diagnostic feature for distinguishing species within the genus *Equisetum*.

The results from the applied DNA barcoding support the taxonomic treatment of the studied *Equisetum* species. The analyzed samples cluster together with several other representatives of the genus *Equisetum* and show the highest degree of homology with sequences of the same species annotated in the NCBI bio-database.

The studied species of *Equisetum* (*E. arvense*, *E. telmateia*, *E. sylvaticum*, *E. palustre*, *E. ramosissimum*) had a similar chemical composition—proteins (9.3–15.9%), carbohydrates (49.8–59.6%), fiber (20.1–31.4%), oils (1.9–3.7%), and mineral substances (12.3–24.0%). *Equisetum* species were high in protein and carbohydrate content, with low glyceride oil, which determined their relatively high nutritional and caloric value (273.9–319.1 kcal/100 g). The studied species had a high content of sterols and phospholipids, and the palmitic acid predominated, ranging from 69.5% (*E. sylvaticum*) to 78.7% (*E. arvense*). On the other hand, low amounts of tocopherols were identified only in *E. palustre* and *E. ramosissimum* with α-tocopherol being predominant. The chemical composition of the studied *Equisetum* species and their biologically active lipid constituents may suggest their suitability for application in the chemical and pharmaceutical industry, in phytotherapy and cosmetics, as well as in organic agriculture as a soil improver.

## Figures and Tables

**Table 1:** Additional anatomical traits for distinguishing the two subgenera.

	**Feature**	**Subgenus**	**Number of Stem Ridges**	**Shape of the Stem Ridges**	**Arrangement of Mechanical Tissue**
**Species**					
*E. arvense*		*Equisetum*	9–10	Well defined	Well-developed in the region of the ridges.
*E. telmateia*		*Equisetum*	Multi-ridged structure	Poorly defined	Poorly developed, several layers in the ridged areas.
*E. sylvaticum*		*Equisetum*	10–14	Well defined, trapezoidal	Multiseriate in the region of the ridges.
*E. palustre*		*Equisetum*	9–10	Well defined, triangular	Well-developed in the region of the ridges.
*E. ramosissimum*		*Hippochaete*	15	Broadly triangular	Multiseriate, follows the contour of the ridges.

**Table 2:** Chemical composition of the studied *Equisetum* species *.

Compounds	Species				
	*E. arvense*	*E. telmateia*	*E. sylvaticum*	*E. palustre*	*E. ramosissimum*
Protein, %	11.6 ± 0.3 ^a^	9.3 ± 0.2 ^b^	15.9 ± 0.1 ^c^	12.3 ± 0.1 ^d^	12.6 ± 0.1 ^e^
Carbohydrate, %	52.0 ± 0.7 ^a^	54.1 ± 0.6 ^b^	59.6 ± 0.4 ^c^	52.7 ± 0.6 ^a^	49.8 ± 0.4 ^d^
Insoluble fibers, %	31.4 ± 0.5 ^a^	20.1 ± 0.1 ^b^	24.4 ± 0.7 ^c^	26.8 ± 0.1 ^d^	22.6 ± 0.8 ^e^
Glyceride oil, %	3.7 ± 0.1 ^a^	2.7 ± 0.1 ^b^	1.9 ± 0.2 ^c^	3.5 ± 0.2 ^a^	2.7 ± 0.2 ^b^
Ash, %	17.7 ± 0.2 ^a^	20.6 ± 0.1 ^b^	12.3 ± 0.0 ^c^	16.9 ± 0.2 ^d^	24.0 ± 0.1 ^e^
Moisture, %	15.0 ± 0.1 ^a^	13.3 ± 0.2 ^b^	10.3 ± 0.1 ^c^	14.6 ± 0.1 ^d^	10.9 ± 0.0 ^e^
Caloric value kcal/100 g, (kJ/100 g)	288.7 ± 0.7 ^a^ 1221.8 ± 3.0	277.9 ± 0.7 ^b^ 1180.4 ± 3.0	319.1 ± 0.6 ^c^ 1355.7 ± 2.5	291.5 ± 0.2 ^d^ 1238 ± 0.9	273.9 ± 3.8 ^b^ 1163.4 ± 16.1

*—All analyses were conducted in triplicate (*n* = 3), and the results are presented as mean ± standard deviation (SD); different letters in the row denote significant differences among examined samples (*p* < 0.05).

**Table 3:** Content and composition of sugars (g/100 g dw) *.

Species	Rhamnose	Fructose	Mannose	Galactose	Sucrose	Total
*E. arvense*	- **	2.00 ± 0.01 ^a^	4.14 ± 0.15 ^a^	- **	- **	6.14 ± 0.16 ^a^
*E. telmateia*	- **	2.00 ± 0.01 ^a^	3.06 ± 0.13 ^b^	- **	1.04 ± 0.02	6.10 ± 0.16 ^a^
*E. sylvaticum*	0.95 ± 0.01 ^a^	0.85 ± 0.01 ^b^	2.52 ± 0.02 ^c^	- **	- **	4.32 ± 0.04 ^b^
*E. palustre*	3.28 ± 0.07 ^b^	2.42 ± 0.08 ^c^	7.61 ± 0.17 ^d^	3.78 ± 0.01 ^a^	- **	17.09 ± 0.33 ^c^
*E. ramosissimum*	- **	3.23 ± 0.08 ^d^	8.20 ± 0.04 ^e^	2.93 ± 0.01 ^b^	- **	14.36 ± 0.13 ^d^

*—All analyses were conducted in triplicate (*n* = 3), and the results are presented as mean ± standard deviation (SD); **—not detected; different letters in the column denote significant differences among examined samples (*p* < 0.05).

**Table 4:** Biologically active substances in the oil and the studied *Equisetum* species *.

Compounds	Species				
	*E. arvense*	*E. telmateia*	*E. sylvaticum*	*E. palustre*	*E. ramosissimum*
Unsaponifiables					
- in oil, %	24.8 ± 0.5 ^a^	15.3 ± 0.2 ^b^	31.1 ± 0.6 ^c^	18.7 ± 0.3 ^d^	16.1 ± 0.3 ^e^
- in the plant, %	0.92 ± 0.04 ^a^	0.41 ± 0.01 ^b^	0.59 ± 0.07 ^c^	0.66 ± 0.05 ^c^	0.43 ± 0.04 ^b^
Sterols					
- in unsaponifiables, %	11.9 ± 0.3 ^a^	8.1 ± 0.1 ^b^	6.2 ± 0.2 ^c^	8.5 ± 0.3 ^b^	14.2 ± 0.2 ^d^
- in oil, %	3.0 ± 0.1 ^a^	1.3 ± 0.1 ^b^	1.9 ± 0.1 ^c^	1.6 ± 0.2 ^b^	2.3 ± 0.2 ^d^
- in the plant, %	0.11 ± 0.01 ^a^	0.04 ± 0.01 ^b^	0.04 ± 0.01 ^b^	0.06 ± 0.01 ^b^	0.06 ± 0.01 ^b^
Tocopherols					
- in oil, mg/kg	- **	- **	- **	82.8 ± 0.8 ^a^	37.6 ± 0.5 ^b^
- α–tocopherol, mg/kgc	- **	- **	- **	82.8 ± 0.8 ^a^	37.6 ± 0.5 ^b^
- in the plant, mg/kg	- **	- **	- **	2.90 ± 0.20 ^a^	1.02 ± 0.09 ^b^
Phospholipids					
- in oil, %	1.6 ± 0.2 ^a^	6.3 ± 0.1 ^b^	11.1 ± 0.2 ^c^	15.3 ± 0.3 ^d^	6.7 ± 0.3 ^b^
- in the plant, %	0.06 ± 0.01 ^a^	0.17 ± 0.01 ^b^	0.21 ± 0.02 ^c^	0.54 ± 0.05 ^d^	0.18 ± 0.02 ^b^

*—All analyses were conducted in triplicate (*n* = 3), and the results are presented as mean ± standard deviation (SD); **—not detected; different letters in the row denote significant differences among examined samples (*p* < 0.05).

**Table 5:** Fatty acid composition of glyceride oils from the studied *Equisetum* species (%) *.

Fatty Acids (FA)		Species				
		*E. arvense*	*E. telmateia*	*E. sylvaticum*	*E. palustre*	*E. ramosissimum*
C_12:0_	lauric	0.5 ± 0.1 ^a^	0.2 ± 0.0 ^b^	0.6 ± 0.1 ^a^	0.3 ± 0.1 ^b^	0.1 ± 0.0 ^c^
C_13:0_	tridecanoic	0.1 ± 0.0 ^a^	- **	0.1 ± 0.0 ^a^	0.1 ± 0.0 ^a^	0.1 ± 0.0 ^a^
C_14:0_	myristic	9.8 ± 0.3 ^a^	5.2 ± 0.2 ^b^	7.5 ± 0.2 ^c^	11.5 ± 0.3 ^d^	0.9 ± 0.1 ^e^
C_14:1_	myristoleic	- **	0.1 ± 0.0 ^a^	- **	0.1 ± 0.0 ^a^	- **
C_15:0_	pentadecanoic	- **	0.5 ± 0.1 ^a^	0.3 ± 0.1 ^b^	0.7 ± 0.2 ^a^	0.4 ± 0.1 ^b^
C_15:1_	pentadecenoic	1.0 ± 0.2 ^a^	0.8 ± 0.1 ^ab^	1.3 ± 0.2 ^a^	0.6 ± 0.1 ^b^	0.6 ± 0.1 ^b^
C_16:0_	palmitic	78.7 ± 0.3 ^a^	80.8 ± 0.4 ^b^	69.5 ± 0.3 ^c^	71.9 ± 0.3 ^d^	70.0 ± 0.2 ^c^
C_16:1_	palmitoleic	- **	- **	- **	0.3 ± 0.0 ^a^	0.4 ± 0.1 ^a^
C_17:0_	margaric	0.3 ± 0.1 ^a^	- **	0.1 ± 0.0 ^b^	0.1 ± 0.0 ^b^	0.1 ± 0.0 ^b^
C_17:1_	heptadecenoic	- **	0.1 ± 0.0 ^a^	- **	0.1 ± 0.0 ^a^	0.2 ± 0.1 ^a^
C_18:0_	stearic	0.2 ± 0.1 ^a^	0.2 ± 0.0 ^a^	0.2 ± 0.0 ^a^	0.4 ± 0.1 ^b^	0.6 ± 0.1 ^b^
trans C_18:1_	elaidic	1.4 ± 0.2 ^a^	0.9 ± 0.1 ^b^	1.5 ± 0.2 ^a^	0.1 ± 0.0 ^c^	0.1 ± 0.0 ^c^
C_18:1_	oleic	1.4 ± 0.1 ^a^	4.6 ± 0.3 ^b^	7.2 ± 0.2 ^c^	3.4 ± 0.1 ^d^	4.5 ± 0.2 ^b^
C_18:2_	linoleic (n-6)	1.6 ± 0.2 ^a^	0.8 ± 0.1 ^b^	1.2 ± 0.1 ^c^	4.5 ± 0.2 ^d^	10.6 ± 0.3 ^e^
C_18:3_	linolenic (n-3)	- **	- **	- **	0.1 ± 0.0 ^a^	0.1 ± 0.0 ^a^
C _20:1_	gadoleic	2.5 ± 0.1 ^a^	2.5 ± 0.1 ^a^	2.1 ± 0.1 ^b^	3.2 ± 0.2 ^c^	9.0 ± 0.3 ^d^
C_22:1_	erucic	1.0 ± 0.1 ^a^	1.4 ± 0.2 ^b^	3.3 ± 0.3 ^c^	1.0 ± 0.2 ^abd^	0.7 ± 0.1 ^d^
C_24:0_	lignoceric	1.5 ± 0.2 ^a^	1.9 ± 0.2 ^a^	5.1 ± 0.1 ^b^	1.6 ± 0.2 ^a^	1.6 ± 0.1 ^a^

*—All analyses were conducted in triplicate (*n* = 3), and the results are presented as mean ± standard deviation (SD); **—not detected; different letters in the row denote significant differences among examined samples (*p* < 0.05).

**Table 6:** Individual sterol composition of oils from studied *Equisetum* species (%) *.

Sterols	Species				
	*E. arvense*	*E. telmateia*	*E. sylvaticum*	*E. palustre*	*E. ramosissimum*
Cholesterol	0.2 ± 0.1	- **	- **	- **	- **
Brassicasterol	0.5 ± 0.2 ^a^	0.4 ± 0.1 ^a^	0.4 ± 0.1 ^a^	0.3 ± 0.1 ^a^	0.1 ± 0.0 ^b^
Campesterol	34.2 ± 0.4 ^a^	27.9 ± 0.3 ^b^	24.1 ± 0.2 ^c^	32.1 ± 0.4 ^d^	37.8 ± 0.5 ^e^
Stigmasterol	0.3 ± 0.0 ^a^	1.2 ± 0.2 ^b^	3.8 ± 0.1 ^c^	0.6 ± 0.1 ^d^	- **
β-Sitosterol	51.1 ± 0.3 ^a^	67.9 ± 0.5 ^b^	69.0 ± 0.4 ^c^	65.3 ± 0.3 ^d^	61.7 ± 0.2 ^e^
Δ^5^-Avenasterol	1.8 ± 0.2 ^a^	0.2 ± 0.0 ^b^	0.2 ± 0.1 ^bc^	0.4 ± 0.1 ^c^	0.2 ± 0.0 ^b^
Δ^7^-Stigmasterol	10.4 ± 0.4 ^a^	2.2 ± 0.2 ^b^	2.2 ± 0.2 ^b^	0.7 ± 0.1 ^c^	- **
Δ^7^-Avenasterol	1.5 ± 0.2 ^a^	0.2 ± 0.1 ^b^	0.3 ± 0.1 ^b^	0.6 ± 0.1 ^c^	0.2 ± 0.0 ^b^

*—All analyses were conducted in triplicate (*n* = 3), and the results are presented as mean ± standard deviation (SD); **—not detected; different letters in the row denote significant differences among examined samples (*p* < 0.05).

**Table 7:** Individual composition of phospholipids fraction of oils from the studied *Equisetum* species (%) *.

Phospholipids	Species				
	*E. arvense*	*E. telmateia*	*E. sylvaticum*	*E. palustre*	*E. ramosissimum*
Phosphatidylcholine	12.4 ± 0.2 ^a^	13.9 ± 0.4 ^b^	21.8 ± 0.5 ^c^	19.6 ± 0.3 ^d^	15.8 ± 0.2 ^e^
Phosphatidylethanolamine	35.9 ± 0.3 ^a^	11.7 ± 0.2 ^b^	11.6 ± 0.1 ^b^	24.3 ± 0.3 ^c^	19.9 ± 0.4 ^d^
Phosphatidylinositol	18.7 ± 0.2 ^a^	23.1 ± 0.3 ^b^	9.2 ± 0.2 ^c^	5.3 ± 0.1 ^d^	12.4 ± 0.3 ^e^
Phosphatidic acids	13.2 ± 0.1 ^a^	26.6 ± 0.2 ^b^	46.0 ± 0.3 ^c^	42.4 ± 0.4 ^d^	30.7 ± 0.3 ^e^
Monophosphatidylglycerol	2.6 ± 0.1 ^a^	9.4 ± 0.2 ^b^	5.9 ± 0.3 ^c^	4.8 ± 0.1 ^d^	8.9 ± 0.3 ^b^
Lysophosphatidylethanolamine	17.2 ± 0.1 ^a^	15.3 ± 0.2 ^b^	5.5 ± 0.1 ^c^	3.6 ± 0.1 ^d^	12.3 ± 0.3 ^e^

*—All analyses were conducted in triplicate (*n* = 3), and the results are presented as mean ± standard deviation (SD); different letters in the row denote significant differences among examined samples (*p* < 0.05).

**Table 8:** List of taxons studied and locality of their collection.

Species	Floristic Region	Locality (Latitude/Longitude and Altitude)
*Equisetum arvense*	Batak, Rhodope Mts	41°94′ N; 24°22′ E/1036 m
*Equisetum telmateia*	Bratsigovo, Rhodope Mts	42°03′ N; 24°37′ E/599 m
*Equisetum sylvaticum*	Peshtera, Rhodope Mts	42°03′ N; 24°31′ E/450 m
*Equisetum palustre*	Batak, Rhodope Mts	41°94′ N; 24°22′ E/1036 m
*Equisetum ramosissimum*	Batak, Rhodope Mts	41°94′ N; 24°22′ E/1036 m

## Data Availability

The original data presented in this study are included in the article. Further inquiries can be directed to the corresponding author.

## Abbreviations

The following abbreviations are used in this manuscript:

dw	Dry weight
FA	Fatty acids
SFA	Saturated fatty acids
UFA	Unsaturated fatty acids
MUFA	Monounsaturated fatty acids
PUFA	Polyunsaturated fatty acids
PA	Phosphatidic acids
DNA	Deoxyribonucleic acid
PCR	Polymerase chain reaction
AOAC	Association of Official Analytical Chemists
HPLC	High performance liquid chromatography
GC	Gas chromatography
FID	Flame ionization detector
FAMEs	Fatty acid methyl esters
POWO	Plants of the World Online
FAO	Food and Agriculture Organization of the United Nations
